# Phenotyping hemodynamic response to veno‐arterial extracorporeal membrane oxygenation in cardiogenic shock

**DOI:** 10.14814/phy2.70961

**Published:** 2026-06-02

**Authors:** Hoong Sern Lim, Dagmar Vondrakova, Mikulas Micek, Petr Ostadal

**Affiliations:** ^1^ Department of Cardiovascular Sciences, College of Medicine and Health University of Birmingham Birmingham UK; ^2^ Department of Cardiology University Hospitals Birmingham NHS Foundation Trust Birmingham UK; ^3^ Department of Cardiology Second Faculty of Medicine, Charles University and Motol University Hospital Prague Czech Republic; ^4^ Department of Physiology First Faculty of Medicine, Charles University Prague Czech Republic

**Keywords:** cardiogenic shock, extracorporeal membrane oxygenation, physiology

## Abstract

Simulation models suggest that veno‐arterial extracorporeal membrane oxygenation (VA ECMO) uniformly results in left ventricular (LV) distension in cardiogenic shock (CS). This study characterized the hemodynamic response to VA ECMO in animal models of CS, and in patients with CS. LV end‐diastolic pressure (LVEDP) and arterial pulse pressure (PP) were assessed in 58 pigs with CS at 2 levels of VA ECMO flows. Pulmonary artery diastolic pressure (PADP), PA pulse pressure (PAPP), and arterial PP data were collected within 2 h of VA ECMO from 128 patients with CS. The animal study identified 3 phenotypes. The centroids of LVEDP and arterial PP changes for Cluster 1 were +4.99 and −1.85 mmHg, Cluster 2 +0.02 and −5.57 mmHg, and Cluster 3 +1.31 and +15.5 mmHg. The clinical study identified 3 phenotypes: “pulsatile” phenotype (26%, highest arterial PP), “low pulsatility” phenotype (52%, low arterial PP and PAPP), and “LV distension” phenotype (22%, elevated PADP). Acute MI was more common in the “LV distension” phenotype. The “low pulsatility” phenotype had a larger RV diameter and tricuspid regurgitation. In summary, this study identified three hemodynamic phenotypes early post‐VA ECMO that were related to the underlying CS etiology and RV function.

## 1 INTRODUCTION

Peripheral (femoral) veno‐arterial extracorporeal membrane oxygenation (VA ECMO) provides effective hemodynamic support in cardiogenic shock (CS). The non‐physiological circulation produced by VA ECMO has unique hemodynamic consequences, particularly on the left ventricle (LV). Simulation studies describe the “(after‐)loading” of the LV that results in the triad of (i) reduced aortic valve opening associated with reduced systemic arterial pulse pressure (PP), (ii) LV distension on echocardiogram, (iii) elevated LV end‐diastolic pressure (LVEDP) or pulmonary artery wedge pressure (PAWP), and pulmonary hypertension on cardiac catheterization (Burkhoff et al., 2015). The consequent pulmonary congestion may be accompanied by respiratory distress and respiratory failure.

However, not all patients on VA ECMO develop this triad of adverse loading (Lim et al., 2017). Truby et al. reported varying degrees of LV distension (with and without need for decompression) in about one in three patients with CS on VA ECMO (Truby et al., 2017). Kalra et al. appeared to contradict the simulation studies, describing VA ECMO “unloading” of the LV in patients with CS (Kalra et al., 2024). In a VA ECMO flow variation study, Saura et al. showed that PAWP did not change in most patients when VA ECMO flow was increased (Saura et al., 2025). Animal studies have also reported comparable “unloading” between ECMO, micro‐axial flow left ventricular assist devices (LVAD) (Yastrebov et al., 2025). An “unloading” effect on VA ECMO was also described in a model of CS with severe aortic stenosis (Ostadal et al., 2022).

Left ventricular filling pressure is a function of inflow into the LV (transpulmonary blood flow into the LV), the compliance of the LV chamber (increase in LV filling pressure per unit increase in LV filling), and LV output (which is related to the interaction between LV contractile function and afterload) (Lim, 2024). As such, it is perhaps unsurprising that VA ECMO could have a variable effect on LV filling pressure, depending on the effect on transpulmonary blood flow and afterload, and the operating LV contractility and chamber compliance.

This study aims to characterize the hemodynamic response to VA ECMO support. Firstly, we examined the hemodynamic changes in response to increasing VA ECMO support in an animal model of CS due to acute myocardial infarction and global myocardial hypoxia. Secondly, we characterized the hemodynamic phenotypes of a cohort of patients with CS on VA ECMO support using clustering analysis of pulmonary artery catheter data following initiation of VA ECMO support. As an exploratory analysis, we evaluated the clinical course and outcomes of the different hemodynamic phenotypes.

## 2 METHODS

### 2.1 Animal study

Data obtained from prior experimental studies in pigs, female swine (*Sus scrofa* domestica, Large White × Landrace crossbreed), aged 4–5 months and with a mean body weight of 60 kg, were obtained from the Institute of Animal Science (Prague, Czech Republic) and used in the studies. The two CS models were:

1. Global myocardial hypoxia (“global hypoxia model”) to produce biventricular failure CS by adjusting mechanical ventilation to precipitate severe hypoxemia in the blood entering the left chambers of the heart and systemic hypoxia, including myocardial hypoxia. Details of the methodology and research ethics approval have been described previously (Ostadal et al., 2018, 2022).
2. Acute LV ischemia by ligating the left anterior descending artery (“LAD ligation model”), leading to isolated left ventricular failure. Details of the methodology and research ethics approval have been described previously (Ostadal et al., 2024).

The induction of cardiogenic shock is associated with the risk of ventricular arrhythmias. VA ECMO was initiated at low flows to allow treatment of the arrhythmias and prevent the loss of the animal. As a result, baseline data without ECMO support were not available in animals, unlike the complete dataset at <1 L/min and 2 L/min. Therefore, hemodynamic parameters (LVEDP and arterial PP) were measured after the development of CS with tissue hypoperfusion at the baseline ECMO flow of < 1 L/min and after increasing ECMO flow to 2 L/min. For consistency, only data from CS experiments with a patent aortic valve and continuous ECMO flow were included in the analysis. Pulmonary artery catheter data were not available from the animal study.

The animal studies were approved by the Charles University First Faculty of Medicine Institutional Animal Care and Use Committee and were performed at the Animal Laboratory, Department of Physiology, First Faculty of Medicine, Charles University in Prague and Na Homolce Hospital, Prague, Czech Republic, in accordance with Act No 246/1992 Coll., for the protection of animals against cruelty. The investigation and protocol conformed to the Guide for the Care and Use of Laboratory Animals published by the United States National Institutes of Health (Publication No. 85‐23, revised 1985).

### 2.2 Clinical study

The second part of this study included consecutive patients with CS due to acute myocardial infarction, myocarditis or end‐stage heart failure who underwent peripheral (femoral) VA ECMO support from January 2013 to April 2025 from a single centre. Patients were excluded if: (i) complete hemodynamic data from pulmonary artery catheterization were not available, (ii) they had concomitant intra‐aortic balloon pump, micro‐axial flow pumps or other forms of “venting” intervention, (iii) ongoing cardiac arrest and tamponade were excluded. The standardization of mechanical circulatory support (MCS) protocols, as previously described (Lim, 2017), included the insertion of pulmonary artery catheters after institution of VA ECMO. This study only included hemodynamic data within 2 h of VA ECMO insertion before any “unloading” intervention such as micro‐axial flow pump support was initiated. Hemodynamic data closest to the 2‐h time point after initiation of VA ECMO were used.

The vasoactive inotrope score (VIS), used to quantify the total doses of inotropes and vasopressors, was calculated as follows (Lim et al., 2021):

$$\begin{array}{cc} \mathrm{VIS}\; & =\text{dopamine}\;\left(\mathrm{\mu g}/\mathrm{kg}/\min\right)+\text{dobutamine}\;\left(\mathrm{\mu g}/\mathrm{kg}/\min\right)\\ & +100\times \text{epinephrine}\;\left(\mathrm{\mu g}/\mathrm{kg}/\min\right)\\ & +100\times \text{norepinephrine}\;\left(\mathrm{\mu g}/\mathrm{kg}/\min\right)\\ & +10\times \text{milrinone}\;\left(\mathrm{\mu g}/\mathrm{kg}/\min\right)\\ & +10,000\times \text{vasopressin}\;\left(\text{units}/\mathrm{kg}/\min\right) \end{array}$$

Change in VIS and lactate clearance was calculated as the change from baseline to 12 hours post‐VA ECMO insertion. In‐hospital outcomes (death, heart transplantation, durable LVAD implant or weaned from MCS) were reported for each group. This study has institutional approval as part of an evaluation of VA ECMO‐related complications, including the use of unloading interventions (CARMS‐17781) (Lim, 2022), and patient consent was waived, but anonymization of data was mandated, such that only aggregated age and sex data were available, date of procedure was removed, and indications for MCS were categorized into broad diagnostic bins.

### 2.3 Statistical analysis

The normal distribution of data was assessed with the Shapiro‐Wilks test. Continuous variables were expressed as median (interquartile range, IQR) for non‐normally distributed or mean ± SD for normally distributed variables. For 2‐group or >2‐group comparisons, we used *t*‐tests and analysis of variance (ANOVA) including post hoc Tukey test for normally distributed data, and Mann–Whitney or Kruskall–Wallis including Dunn tests for non‐normally distributed data. Chi‐squared cross tabulation was used to compare categorical data between groups. Bonferroni correction was used for multiple comparisons and *p* values of <0.05 were considered statistically significant.

K‐means clustering iteratively enforces each data point into distinct, non‐overlapping subgroups (clusters). The selection of hemodynamic features for K‐means clustering was based on (i) physiological considerations (transpulmonary blood flow, aortic valve opening and LV distension), to preserve the clinical interpretability of the resulting clusters, and (ii) the degree of correlation between the features (bias associated with highly correlated variables). One of any pair of variables that were highly correlated (*r* > 0.8) was removed which obviated the need for principal component analysis. The elbow method (semi‐quantitatively) and the maximum silhouette score were used to identify the optimal number of clusters. Both the Davies–Bouldin Index and Calinski–Harabasz Index were used to evaluate the partitioning of the clusters. All data were normalized to account for potential differences in the scale (StandardScaler from sklearn) prior to clustering analysis. After separation into clusters, the cluster assignments were used as labels and a random forest classifier (RandomForestClassifier from sklearn) was used to predict which cluster each patient belonged to, based on the selected hemodynamic features, as a form of post hoc validation. The centroid refers to the vector of feature means and as K‐means clustering is distance‐based, the centroid is mathematically the point of minimum sum of squared distances within the cluster.

For the animal study, we used the changes in LVEDP and arterial PP as the features for the clustering analysis, not the static values of LVEDP or arterial PP. Therefore, the clusters in the animal study characterized the direction of change in LVEDP and PP. The features used for the animal study differed from the clinical study, which used post‐VA ECMO hemodynamic parameters as features for the clustering analysis.

All analyses were performed on Python (v13.12, Python Software Foundation, software packages included Sci‐kit‐learn, Scipy, Pandas, Numpy, Seaborne, and Matplotlib).

## 3 RESULTS

### 3.1 Animal study

The experimental study included 58 animals (LAD ligation, *n* = 30, global hypoxia, *n* = 28). After induction of CS and on baseline VA ECMO flow of <1 L/min, the LVEDP and arterial PP were higher in the LAD ligation compared to the global hypoxia model (Data S1). Increasing VA ECMO flow from <1 L/min to 2 L/min was associated with an increase in LVEDP from 13.8 ± 4.7 to 14.6 ± 5.7 mmHg (*p* = 0.011) and a decrease in arterial PP from baseline 29.0 ± 13.2 to 25.3 ± 12.3 mmHg (*p* < 0.001). The change in LVEDP and arterial PP were not significantly different between the LAD ligation (0.4 (−0.05–2.0) mmHg, *p* = 0.260) and global hypoxia (0.06 (−0.5–1.0) mmHg, *p* = 0.261) models.

**FIGURE 1:**
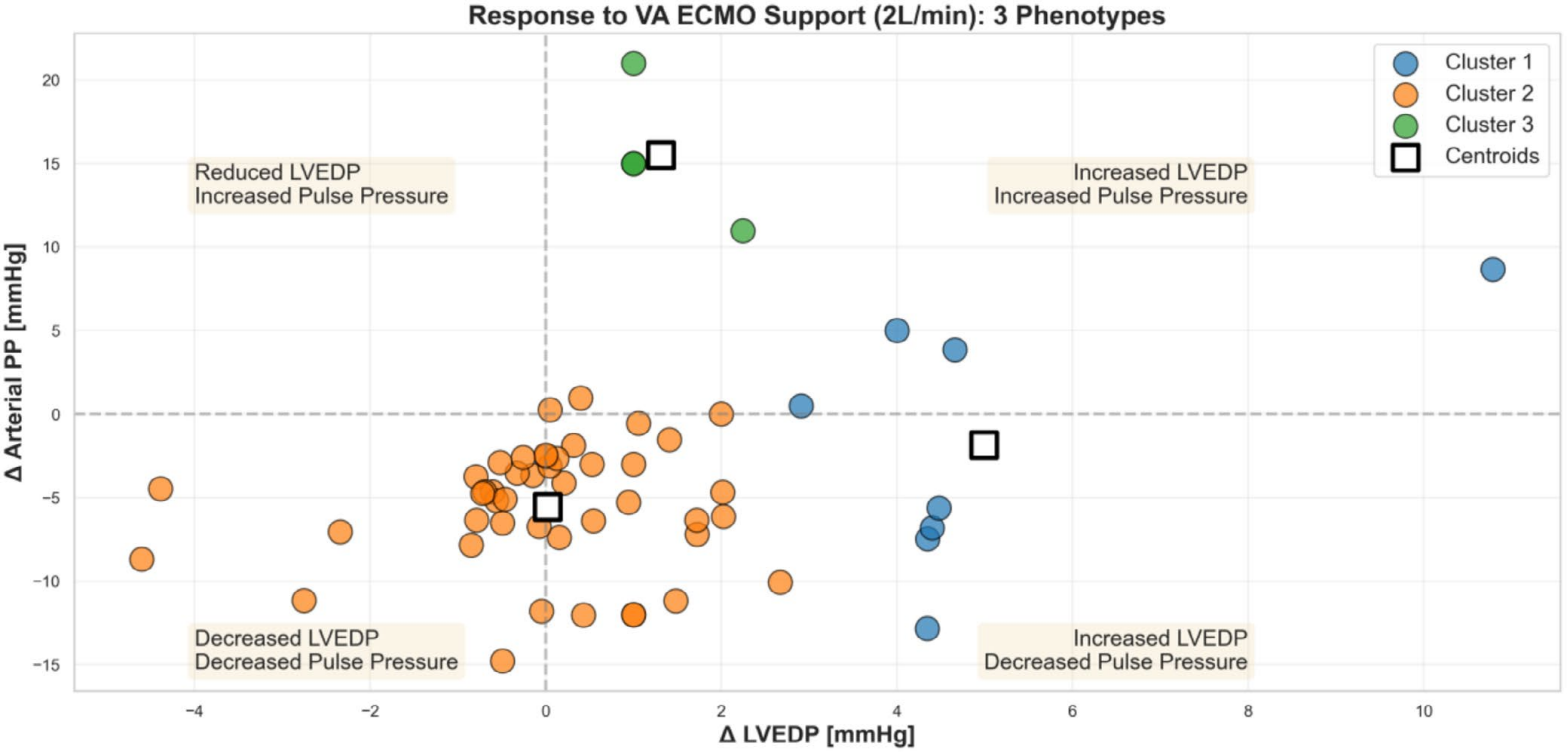
Changes in LVEDP and arterial PP when VA ECMO flows increased from <1 L/min to 2 L/min in 58 pigs.

However, the response to increased VA ECMO flow was not uniform. Using K‐means clustering, we were able to identify 3 clusters that demonstrated different changes in LVEDP and arterial PP (Figure 1). Baseline LVEDP (14.4 (9.7–16.3) versus 13.5 (10.2–16.0) versus 17.5 (17.0–18.8) mmHg, *p* = 0.061) and arterial PP (30.9 (23.7–37.0) versus 30.9 (24.3–36.1) versus 19.5 (9.5–29.3) mmHg, *p* = 0.473) were comparable between the 3 clusters. The centroids for the changes in LVEDP and arterial PP for Cluster 1 (*n* = 8, 14%) were + 4.99 and −1.85 mmHg, Cluster 2 (*n* = 45, 79%) were +0.02 and −5.57 mmHg, and Cluster 3 (*n* = 4, 7%) were +1.31 and +15.5 mmHg.

### 3.2 Clinical study

A total of 179 patients fulfilled the inclusion and exclusion criteria, of which 128 patients had the complete hemodynamic data to be included for this study (CONSORT diagram and the baseline characteristics of the 128 patients with CS in Data S1). Hemodynamic data were recorded 110 ± 20 min from the initiation of VA ECMO. Of the 128 patients, 118 patients (92%) were mechanically ventilated following VA ECMO. Advanced HF was the dominant cause of CS, but AMI was more common in males and acute myocarditis more common in females (Data S1). Baseline blood pressure, lactate, and VIS were comparable between males and females.

**FIGURE 2:**
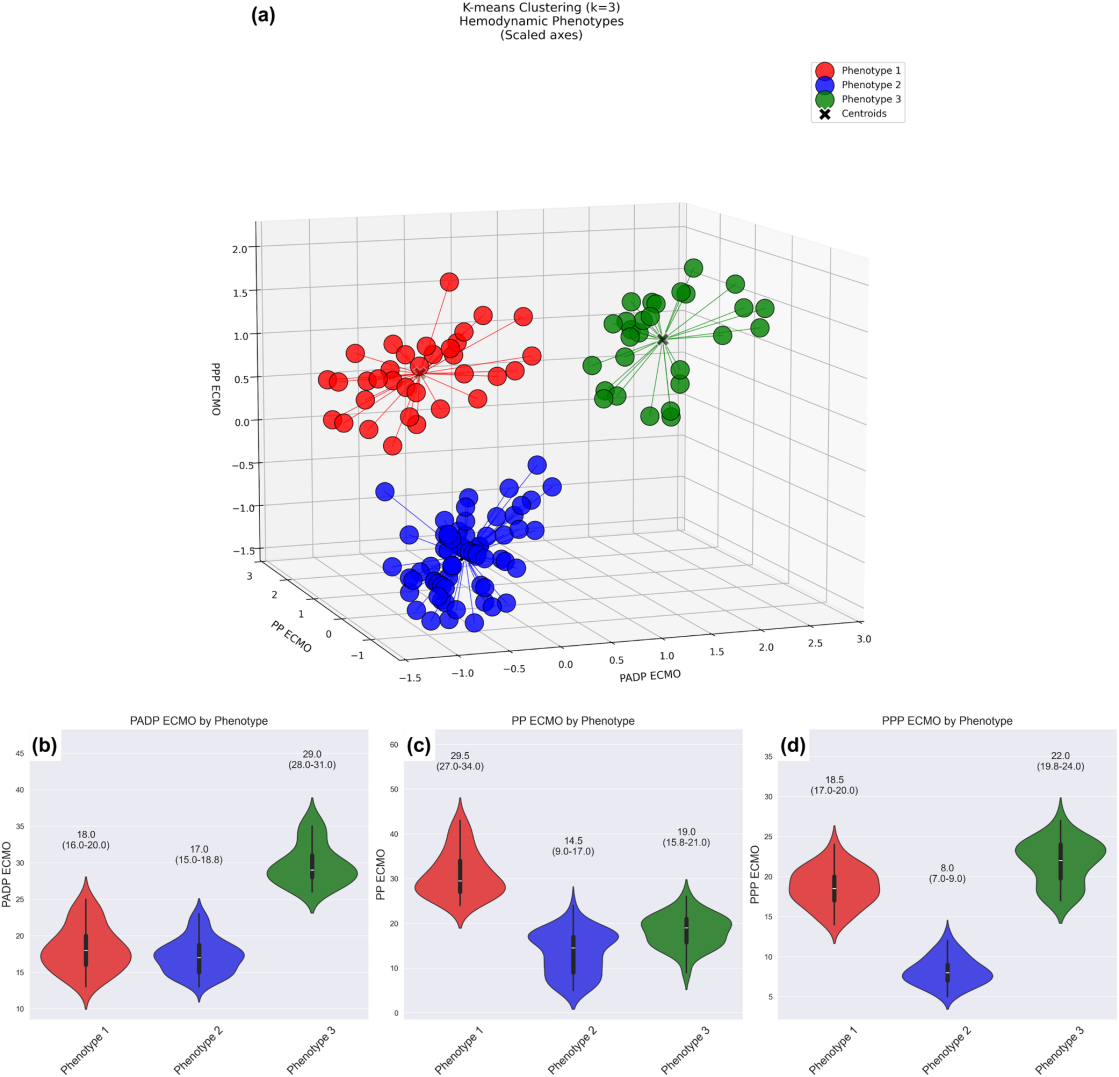
Pulmonary artery PP (PPP ECMO), arterial PP (PP ECMO), and pulmonary artery diastolic pressure (PADP ECMO) on VA ECMO support for the three clusters. A scaled scatter plot of the hemodynamic data for the three phenotypes (a). Violin plots to compare the hemodynamic data for the three hemodynamic phenotypes based on pulmonary artery diastolic pressure (b), arterial PP (c), and pulmonary artery PP (d) (all *p* < 0.001).

Clustering analysis identified three clusters (maximum silhouette score of 0.61) – Phenotypes 1, 2, and 3 – based on the post‐VA ECMO PA PP, arterial PP and PA diastolic pressure (Figure 2a, Data S1). Phenotype 1 (*n* = 34, 26%) had the highest arterial PP, that is, the “pulsatile” phenotype. Phenotype 2 (*n* = 66, 52%) was characterized by low systemic arterial and PA PP, that is, “low pulsatility” phenotype. Phenotype 3 (*n* = 28 or 22%) had markedly elevated PADP, that is, the “LV distension” phenotype (Figure 2b). The “LV distension” phenotype was more common in males (25 (27.5%) vs. 3 (8.1%), Data S1) but the difference was not significant. Post‐VA ECMO hemodynamic data and the correlation matrix are shown in Data S1. The VA ECMO flows were lowest in the “LV distension” Phenotype 3 compared to “pulsatile” and “low pulsatility” phenotypes (4.04 ± 0.40 vs. 4.17 ± 0.42 vs. 4.27 ± 0.40, *p* = 0.038), even when indexed to body surface area (2.0 (1.9–2.2) vs. 2.2 (2.0–2.3) vs. 2.2 (2.1–2.3) L/min/m^2^, Bonferroni‐corrected *p* < 0.05). The product of MAP and VA ECMO flow was highest in the “pulsatile” compared to the “low pulsatility” and “LV distension” phenotypes (294 (270–314) vs. 267 (252–284) vs. 269 (258–289), *p* = 0.001).

### 3.3 Differences in characteristics between hemodynamic phenotypes

There were significant differences in baseline characteristics between the three hemodynamic phenotypes (Table 1). Among patients with “low pulsatility” Phenotype 2, CS was predominantly related to advanced HF, in contrast to the preponderance of AMI as the cause of CS in the “LV distension” Phenotype 3. Patients with the “low pulsatility” Phenotype 2 also had more severe RV impairment (larger RV diameter and lower tricuspid annular plane systolic excursion and a larger proportion with severe tricuspid regurgitation), which was accompanied by liver and renal dysfunction.

**TABLE 1:** Baseline (Pre‐VA ECMO) characteristics of the three phenotypes.

Variable	Phenotype 1 (“pulsatile”) (*n* = 34)	Phenotype 2 (“low pulsatility”) (*n* = 66)	Phenotype 3 (“LV distension”) (*n* = 28)	*p*
Indication (*n*, %)				<0.001
Advanced HF	20 (58%)	51 (77%)	11 (39%)	
AMI	7 (21%)	5 (8%)	14 (50%)	
Acute myocarditis	7 (21%)	10 (15%)	3 (11%)	
Prior arrest (*n*, %)	7 (21%)	13 (20%)	4 (14%)	0.786
Creatinine (μmol/L)	151 (130–163)	168 (140–199)	105 (91–129)^*^	<0.001
Bilirubin (μmol/L)	32 (24–53)	61 (45–69)^*^	29 (20–36)	<0.001
LVEF (%)	17 (14–21)	15 (11–19)^*^	20 (15–25)	0.005
LVEDD (cm)	5.6 ± 0.9	5.8 ± 1.1	5.6 ± 0.8	0.541
TAPSE (mm)	12.6 ± 1.7	10.7 ± 1.9^*^	13.3 ± 2.0	<0.001
RVD (cm)	3.3 (2.9–3.5)	3.9 (3.6–4.4)^*^	3.1 (2.9–3.5)	<0.001
Severe MR (*n*, %)	7 (21%)	17 (26%)	4 (14%)	0.345
Severe TR (*n*, %)	1 (3%)	18 (27%)^*^	2 (7%)	<0.001
Heart rate (bpm)	93 ± 8^*^	102 ± 12^*^	108 ± 13^*^	<0.001
SBP (mmHg)	83 ± 5	83 ± 5	83 ± 5	0.673
DBP (mmHg)	54 ± 3	51 ± 5^*^	56 ± 4	<0.001
MAP (mmHg)	67 ± 3	66 ± 4	68 ± 4^*^	0.014
Lactate (mmol/L)	5.9 (4.4–10.3)	6.2 (4.3–9.1)	6.2 (5.4–8.9)	0.762
Vasoactive inotrope score	21.7 (17.0–28.4)	19.6 (17.0–25.1)	23.1 (15.1–27.0)	0.370

Abbreviations: AMI, acute myocardial infarction; bpm, beats per minute; DBP, diastolic blood pressure; HF, heart failure; LVEDD, left ventricular end‐diastolic diameter; LVEF, left ventricular ejection fraction; MAP, mean arterial blood pressure; MR, mitral regurgitation; RVD, right ventricular diameter; SBP, systolic blood pressure; TAPSE, tricuspid annular plane systolic excursion; TR, tricuspid regurgitation.

^*^ *p* < 0.05.

There were significant reductions in VIS and lactate in all three phenotypes at 12 hours post‐VA ECMO, but the magnitude of change differed between groups. The percentage reduction in VIS was significantly lower in the “low pulsatility” phenotype 2 compared to phenotypes 1 and 3 (35 (19–50) vs. 57 (51–70) and 59 (40–65) %, Bonferroni‐corrected *p* < 0.001). Lactate clearance, expressed as percentage reduction from baseline, was significantly higher in “pulsatile” phenotype 1 compared to phenotypes 2 and 3 (58 (41–70) vs. 48 (37–54) vs. 43 (32–56) %, Bonferroni‐corrected *p* < 0.05).

**FIGURE 3:**
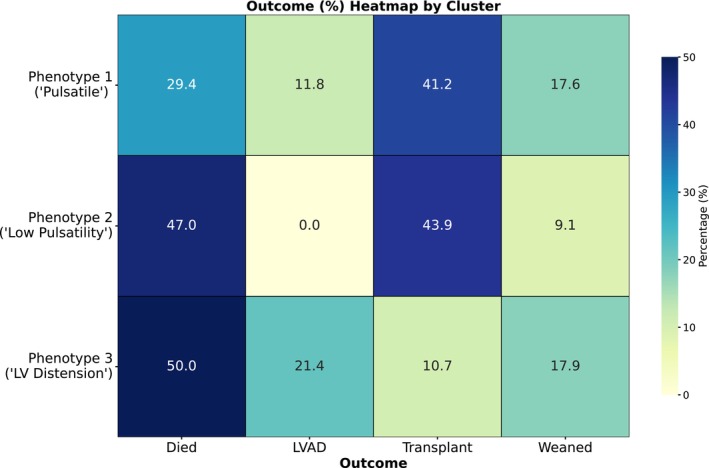
In‐hospital outcomes based on the hemodynamic phenotype on VA ECMO.

**FIGURE 4:**
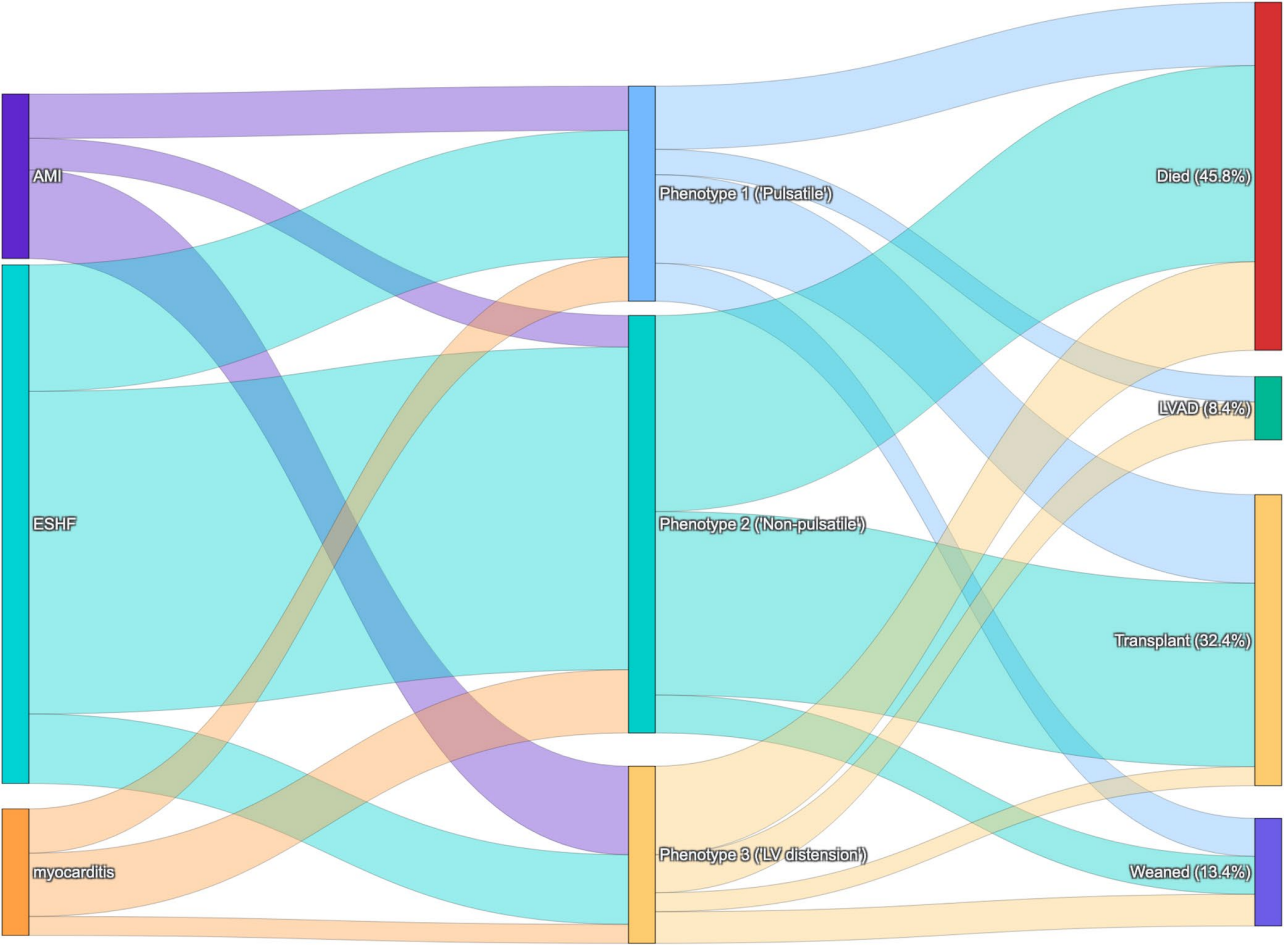
A Sankey diagram from the underlying etiology to the phenotype and outcomes. In‐hospital mortality was lowest in Phenotype 1 and significant differences in durable LVAD and heart transplantation between phenotypes (Bonferroni‐corrected *p* < 0.05).

Overall, in‐hospital mortality was 46%. In‐hospital outcomes varied by the etiology and hemodynamic phenotype (Figure 3). Patients with HF‐related CS were significantly more likely to undergo heart transplantation, but significantly higher proportion of patients with acute myocarditis were liberated from MCS (all *p* < 0.05 after Bonferroni correction). In‐hospital mortality was significantly lower in the “pulsatile” Phenotype 1. Both Phenotypes 1 and 2 were more likely to undergo heart transplantation, while patients of the “LV distension” Phenotype 3 were significantly more likely to undergo durable LVAD implantation (all *p* < 0.05 after Bonferroni correction). The outcomes based on the etiology of CS and the hemodynamic phenotypes are shown in Figure 4.

## 4 DISCUSSION

**FIGURE 5:**
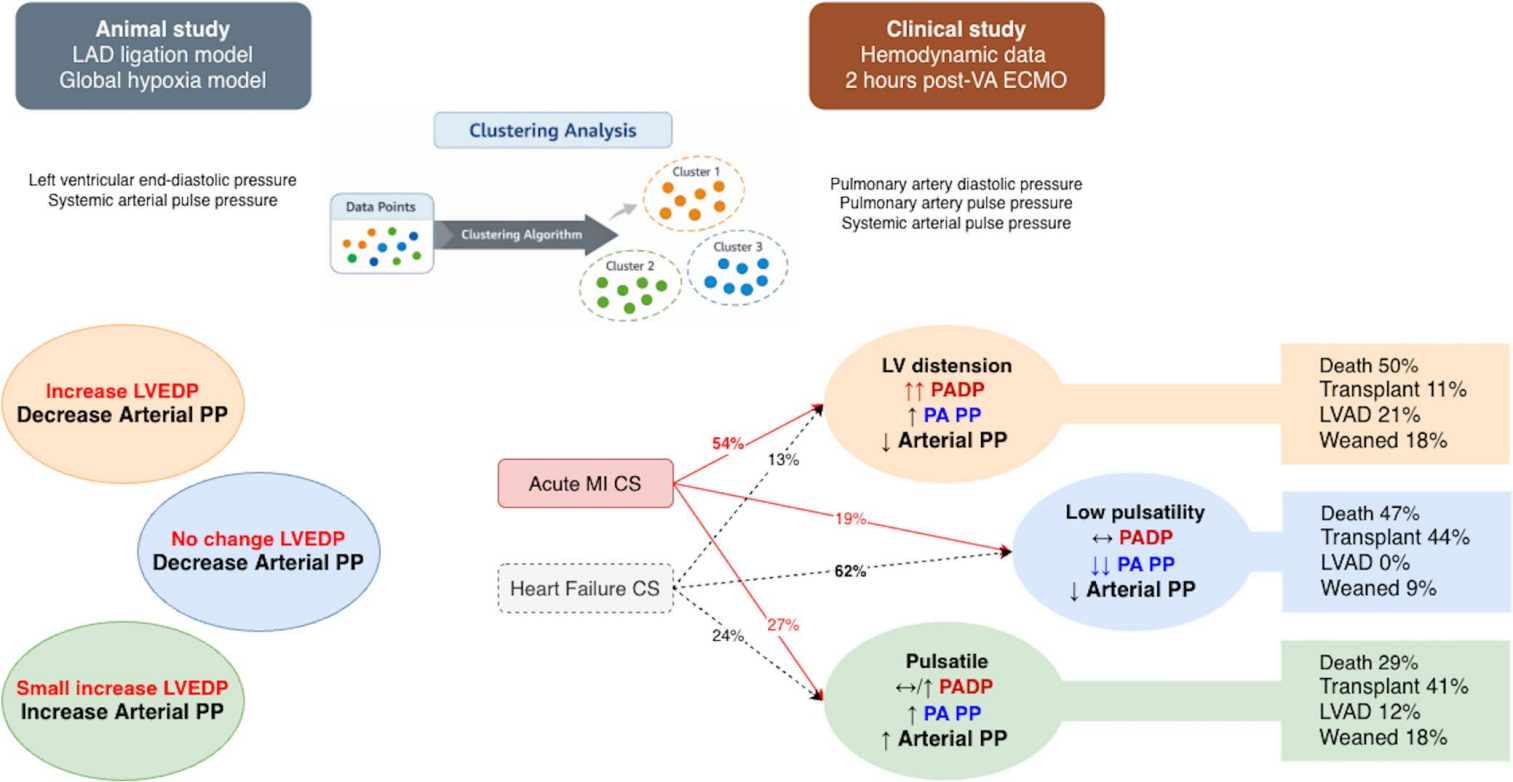
Clustering analysis of the animal study identified 3 clusters based on the changes in LVEDP and arterial PP in response to increase in VA ECMO flows. These 3 clusters corresponded to the 3 post‐VA ECMO hemodynamic phenotypes identified in the clinical study based on post‐VA ECMO PADP, PA PP and systemic arterial PP (“LV distension,” “low pulsatility,” and “pulsatile” phenotypes). Patients with AMI‐ and HF‐related CS were more likely to develop the “LV distension” and “low pulsatility” phenotypes respectively. Outcomes differed between the hemodynamic phenotypes.

In this study, we (i) examined the changes in LVEDP and arterial PP from animal models of CS and increasing VA ECMO support, and (ii) characterized the post‐VA ECMO hemodynamic profiles of a cohort of patients with CS who underwent VA ECMO support without unloading. The animal data showed that the changes in arterial PP and LVEDP varied and the animals could be divided into 3 clusters based on the pattern of changes in arterial PP and LVEDP. In the clinical study, the post‐VA ECMO systemic arterial PP, PA PP, and PADP varied, and 3 clusters, or “hemodynamic phenotypes” could be identified based on these 3 hemodynamic parameters – “pulsatile”, “low pulsatility”, and “LV distension” phenotypes. The clinical course and outcomes differed between the “hemodynamic phenotypes” (Figure 5).

The findings of the animal studies complemented the clinical study. The 3 patterns of changes in arterial PP and LVEDP in the animal data corresponded to the 3 “hemodynamic phenotypes” in the clinical study. Cluster 1 of the animal study showed an increase in LVEDP and reduction in arterial PP, from which a “LV distension” phenotype could be inferred. Cluster 2 showed no change in LVEDP but a reduction in arterial PP, suggestive of the “low pulsatility” phenotype, and Cluster 3, with a small increase in LVEDP and larger increase in arterial PP, would be consistent with the “pulsatile” phenotype.

This study stemmed from our empirical observations of heterogeneous hemodynamic response to VA ECMO, in contrast to simulation models that uniformly described progressive rightward/upward displacement and narrowing of the pressure‐volume loop, that is, increasing LVEDP and diminishing LV stroke volume. In our animal experiments, 35% of cases showed reduction in LVEDP when VA ECMO flow was increased. Our data in patients with CS showed that the “LV distension” phenotype only developed in the minority of cases, albeit over a short 2‐h window. It is possible that a greater proportion of patients could have evolved into the “LV distension” phenotype over a longer period of VA ECMO support. Such dynamic evolution of the hemodynamic phenotype could not be evaluated at our centre due to the widespread use of micro‐axial flow pumps to “unload” the LV.

We selected the features for our clustering analysis based on our hypothesis that, at any level of VA ECMO flow, the changes in LV filling pressure (PADP as surrogate) are related to transpulmonary blood flow (PA PP as surrogate), aortic valve opening (arterial PP as surrogate of LV stroke volume at the prevailing afterload). Indeed, a computational model showed that LV dysfunction alone is insufficient in increasing pulmonary venous pressure. Translocation of blood volume from the systemic venous system into the pulmonary circulation is a necessary physiological mechanism for pulmonary congestion (Magder et al., 2009)– a process that involves vasoconstriction‐mediated transfer of blood volume from “unstressed” to “stressed” compartment and RV function to generate flow to the left heart system.

Our observations were consistent with this hypothetical model. The higher PADP in patients of the “LV distension” phenotype was associated with higher TAPSE, smaller RV diameter, and higher PA PP, indicative of better RV function and higher transpulmonary blood flow, in the setting of reduced LV emptying (small amplitude arterial PP). In contrast, the smaller arterial PP amplitude in the “low pulsatility” phenotype was not associated with LV distension, probably because of the reduced transpulmonary blood flow associated with poorer RV function (small amplitude PA PP).

The differences in the underlying etiology of CS may also be relevant. Filling pressure is related to the intrinsic filling properties of the heart that is defined by the slope of the diastolic pressure‐volume relationship. Low compliance of the LV chamber produces a steep slope such that the same increase in LV volume may produce a greater increase in filling pressure. Acute MI results in “stiffer” less compliant LV (Diamond & Forrester, 1972). We postulate that LV distension in response to VA ECMO is more likely in acute MI, as higher transpulmonary blood flow due to better RV function coupled with “stiffer,” less compliant LV in the setting of acute ischemia is more likely to produce greater elevations in LVEDP. This may explain our observation that 54% of patients with acute MI developed the “LV distension” phenotype in contrast to 13% of patients with HF‐related CS. Our observation of higher LVEDP in the LAD ligation animal model at baseline and on higher VA ECMO flow, compared to the global hypoxia model, is also consistent with the clinical study.

Reduction in arterial PP is usually interpreted as reduction in LV stroke volume. Mourad et al. (2020). showed that a PP <15 mmHg correlated strongly with reduced native cardiac output (<1 L/min). Our animal data showed that reduction in arterial PP is common when VA ECMO flow was increased. From our data in patients with CS on VA ECMO, we could infer that the small amplitude arterial PP may be related to two mechanisms: reduced transpulmonary flow and reduced preload (i.e. “low pulsatility” Phenotype 2) or LV afterload mismatch (i.e. “LV distension” Phenotype 3). A combination of varying degrees of LV preload reduction and afterload mismatch could be at play to reduce native cardiac output and arterial PP, but it is not possible to quantify the relative contributions of these two mechanisms on the LV in this study.

Small amplitude arterial PP on VA ECMO has also been reported to be associated with reduced survival (Schmidt et al., 2015). In our cohort of patients with CS, phenotypes with lower arterial PP similarly had higher in‐hospital mortality compared to the “pulsatile” Phenotype 1. However, outcomes were inextricably linked to the underlying etiology. It is difficult to determine if the higher in‐hospital mortality associated with smaller amplitude arterial PP is related to the hemodynamic effects of VA ECMO or the underlying cause of CS. Of note, the clinical course and outcomes varied with the hemodynamic phenotypes, but these findings should be considered exploratory only and will require further study, as these results could be confounded by numerous factors such as the management of MCS and institutional approach to heart replacement therapy.

The clinical implication of our study is twofold. Firstly, the randomized trial of VA ECMO in CS due to acute myocardial infarction has failed to demonstrate a reduction in mortality (Thiele et al., 2023). The heterogeneous hemodynamic response to VA ECMO may explain the limitations of applying a single MCS modality to a diverse group of patients with a highly heterogenous response to VA ECMO. Understanding the potential hemodynamic response to VA ECMO may facilitate the tailoring of MCS strategy. In the face of such heterogeneity, the use of pulmonary artery catheters to characterize the hemodynamic profile of patients with CS may be of benefit (Garan et al., 2020), especially in guiding the management of MCS (Bernhardt et al., 2023).

Secondly, these data may have implications on the use of LV unloading interventions. Early studies suggested that the additional use of micro‐axial flow pumps to unload the LV during VA ECMO support may improve survival in patients with CS (Pappalardo et al., 2017). These early findings were supported by larger multi‐center registry studies that suggested that LV unloading may reduce mortality in patients with CS treated with VA ECMO (Russo et al., 2019), at the cost of significant complications (Schrage et al., 2020). Subsequent registry data further suggested that early unloading (defined by implantation before up to 2 h after VA ECMO) may increase the likelihood of weaning from ventilation and lower risk of 30‐day mortality (Schrage et al., 2023). Trials are ongoing to evaluate LV unloading with micro‐axial pumps in patients on VA ECMO (NCT05577195). Our data suggest that early post‐VA ECMO hemodynamic evaluation to guide judicious use of LV unloading strategies, tailored to the hemodynamic phenotype, may maximize the risk‐versus‐benefit considerations in patients with CS on VA ECMO support.

### 4.1 Study limitations

This study suffers from several limitations. First, as an observational study, this study is susceptible to biases. The patients included in this study were highly selected, with the exclusion of active cardiopulmonary resuscitation and other causes of CS. Patient selection for VA ECMO and practice changes over the course of this study may introduce biases, and there may be measured and unmeasured differences in patients with missing data compared to the cohort in this study. In particular, the clinical course and outcomes of the phenotypes would be confounded by differences in clinical management, and must be interpreted with caution and considered preliminary. Our findings should be validated in larger cohorts of patients on VA ECMO. Secondly, we used PADP as a surrogate for PAWP, based on the assumption that significant pulmonary vascular disease is absent. Thirdly, this study only captured early (<2 h) hemodynamic response to VA ECMO. Hemodynamic parameters could change during the course of VA ECMO support in response to changing volume status, vasoactive drug use and changes in native cardiac function. Future studies should evaluate the longitudinal evolution of hemodynamic profiles on VA ECMO. Fourthly, the heterogeneity in response to VA ECMO was demonstrated in our animal data, but we were unable to assess the pre‐ and post‐VA ECMO changes in hemodynamic parameters in our cohort of patients with CS because pre‐VA ECMO hemodynamic data were limited.

## 5 CONCLUSION

Increasing VA ECMO flow has different effects on the LV. We described three hemodynamic phenotypes –“pulsatile,” “low pulsatility,” and “LV distension.” These hemodynamic phenotypes on peripheral VA ECMO were related to the underlying etiology and pathophysiology of CS. The implications on clinical outcomes need further study.

## AUTHOR CONTRIBUTIONS

**Hoong Sern Lim:** Conceptualization; data curation; formal analysis; investigation; methodology; project administration; resources; software; validation; visualization. **Dagmar Vondrakova:** Data curation; investigation. **Mikulas Micek:** Data curation; formal analysis; investigation; methodology. **Petr Ostadal:** Conceptualization; data curation; formal analysis; funding acquisition; investigation; resources; supervision.

## FUNDING INFORMATION

The authors have nothing to report.

## CONFLICT OF INTEREST STATEMENT

The authors declare no conflicts of interest.

## ETHICS STATEMENT

The animal studies were approved by Charles University First Faculty of Medicine Institutional Animal Care and Use Committee.

## Supporting information


Data S1.

## Data Availability

The data that support the findings of this study are available from the corresponding author upon reasonable request.
