# Supplementary material for: Phenotyping hemodynamic response to veno‐arterial extracorporeal membrane oxygenation in cardiogenic shock
Source: Physiol Rep. 2026 Jun 2;14(11):e70961. doi: 10.14814/phy2.70961 (PMC13239767; doi:10.14814/phy2.70961)

Supplementary Data

**Phenotyping hemodynamic response to veno-arterial extracorporeal membrane oxygenation in cardiogenic shock**

1. Hemodynamic data in animal study
2. CONSORT flow diagram
3. Demographics and baseline data
4. Correlation matrix and stability of K-means clustering
5. Hemodynamic parameters on VA ECMO by hemodynamic phenotypes
6. In-hospital outcomes by etiology
7. In-hospital outcomes by hemodynamic physiology
8. Baseline and hemodynamic data by sex
9. 9. A box plot of ECMO power (the product of VA ECMO flow and MAP) for the three hemodynamic phenotypes.

1. Hemodynamic data in animal study.

Changes in LVEDP and arterial PP from baseline (<1L/min) to 2L/min of VA ECMO flow in the LAD ligation and global hypoxia models. In the LAD ligation model, LVEDP increased from 14.6 (13.0-17.1) to 15.4 (13.9-19.3) mmHg, and arterial PP decreased from 35.4 (33.1-43.4) to 32.0 (26.7-36.0) mmHg. In the global hypoxia model, LVEDP decreased from 10.5 (9.2-14.7) to 10.1 (9.1-18.1) mmHg, and arterial PP decreased from 24.9 (17.3-27.2) to 20.5 (18.2-24.5) mmHg.


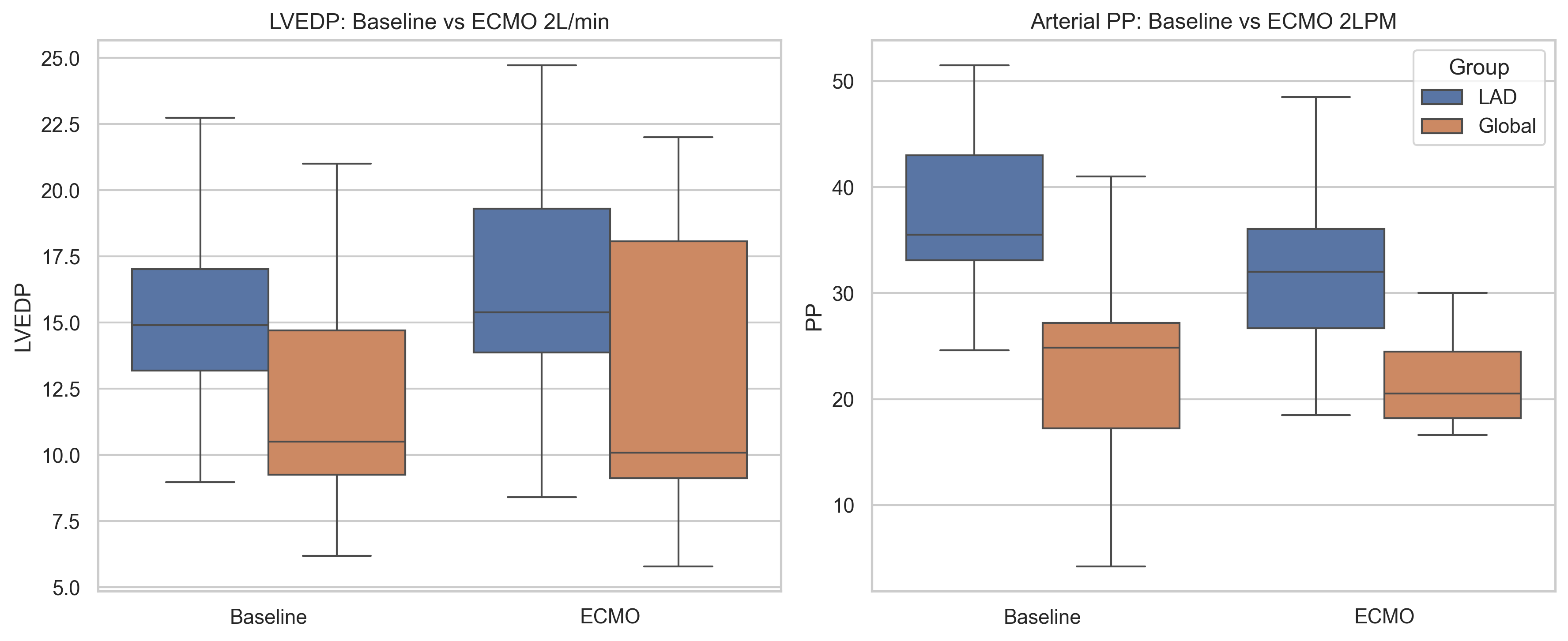


2: CONSORT flow diagram


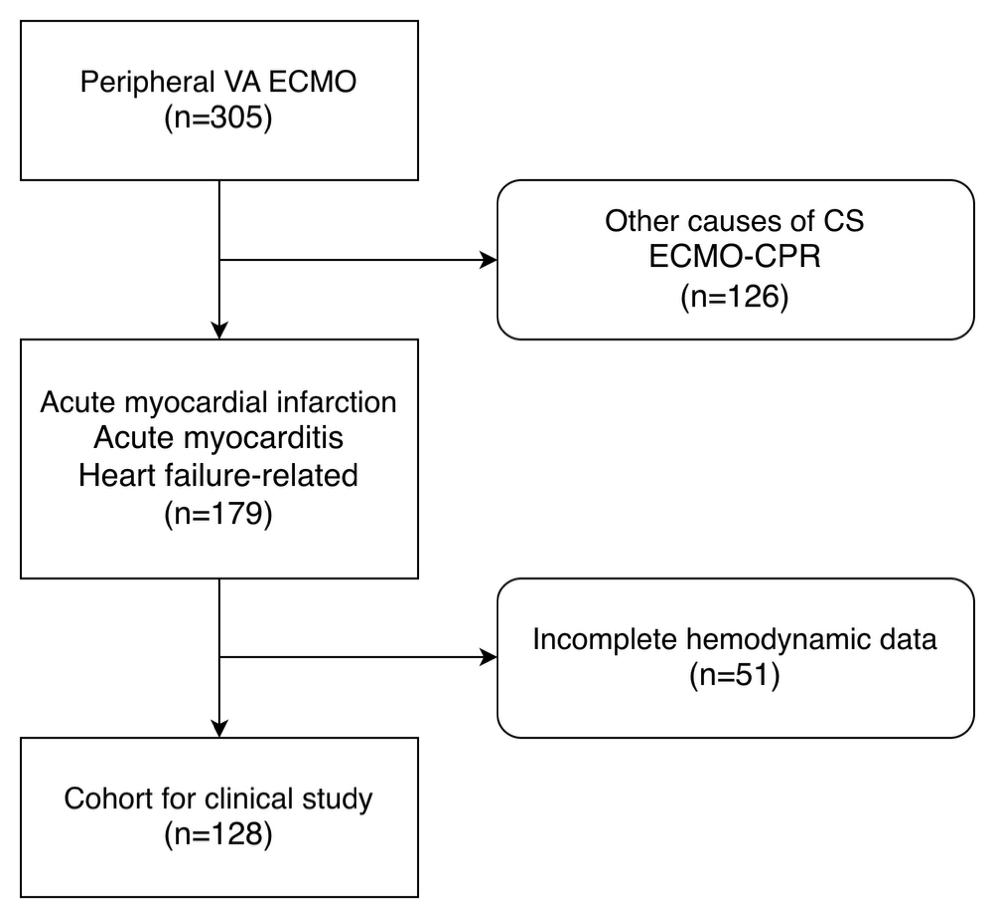


3. Demographics and baseline data (n=128)

| Age > 50 years (n, %) | 64 (50%) |
| --- | --- |
| Males (n, %) | 91 (71%) |
| BMI (kg/m2) | 26.4 ± 4.0 |
| Indication (n, %)   - Advanced heart failure   - Ischemic   - Non-ischemic - Acute myocardial infarction - Acute myocarditis | 82 (64%)  26 (32%)  56 (68%)  26 (20%)  20 (16%) |
| Prior cardiac arrest (n, %) | 24 (19%) |
| Creatinine (umol/L) | 149 (127-181) |
| Bilirubin (umol/L) | 45 (31-63) |
| LV ejection fraction (%) | 16 (12-20) |
| LV end-diastolic diameter (cm) | 5.6 ± 1.0 |
| TAPSE (mm) | 11.7 ± 2.2 |
| Mid-RV diameter (cm) | 3.6 (3.2-3.9) |
| Severe MR (n, %) | 28 (22%) |
| Severe TR (n, %) | 21 (16%) |
| Heart rate (bpm) | 101 ± 13 |
| Systolic BP (mmHg) | 83 ± 5 |
| Diastolic BP (mmHg) | 53 ± 5 |
| Epinephrine (n (%), mcg/kg/min) | 80 (63%), 0.06 (0.04-0.08) |
| Norepinephrine (n (%), mcg/kg/min) | 119 (93%), 0.11 (0.08-0.14) |
| Vasoactive inotrope score | 20.5 (16.5-26.9) |
| Lactate (mmol/L) | 6.2 (4.5-9.3) |

BMI: body mass index; BP: blood pressure; bpm: beats per minute; LV: left ventricular; MR: mitral regurgitation; RV: right ventricular; TAPSE: tricuspid annular plane systolic excursion; TR: tricuspid regurgitation

4: Correlation matrix and stability of K-means clustering

K-means clustering is distance-based and susceptible to bias if the features are highly correlated. One of any pair of variables that were highly correlated (r>0.8) were removed. The correlation matrix is shown below.


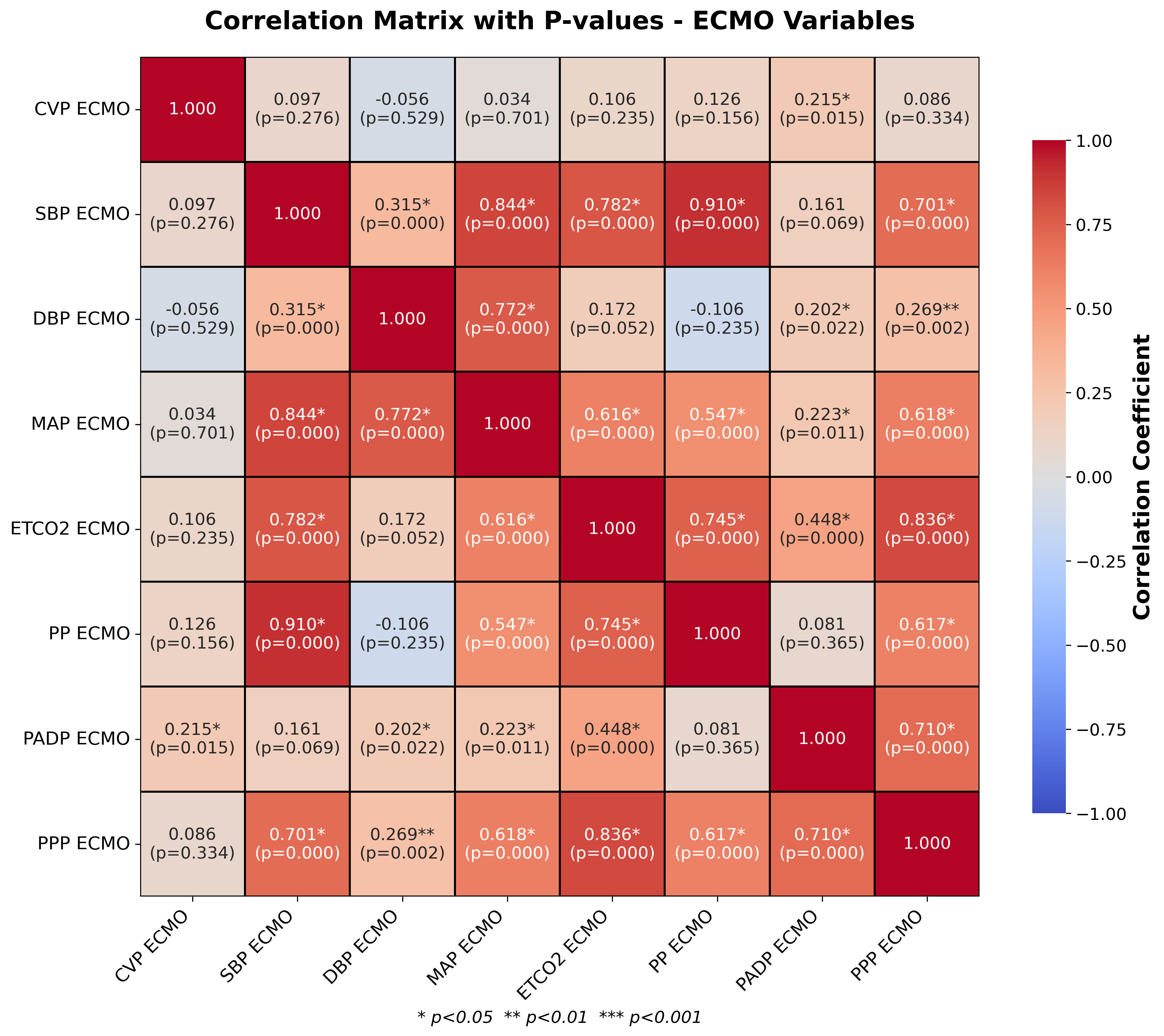


The cluster prediction accuracy from random forest classification was 0.86±0.04, which indicated that random forest correctly predicted the cluster a patient belonged to, based on their three hemodynamic values, 86% of the time.

To assess the robustness of cluster assignments under missing data, we conducted ten stochastic imputations of simulated missing values in the subset of 50 cases with complete data. Cluster stability, defined as the proportion of patients assigned to the same cluster across all ten imputations, was moderately robust at 70% (i.e.: cluster assignments were identical in all ten imputations). The cluster assignment of the 15 cases that varied across the ten imputations are shown below. The majority (73%) of the ‘switched’ clusters were between Phenotypes 1 and 2. Thus, most cluster assignments were stable, with moderate-high stability overall, but a subset of patients showed sensitivity to missing data, especially between Phenotypes 1 and 2. The internal metrics, Davies-Bouldin Index of 0.61 and Calinski-Harabasz Index of 278, also suggest moderate-strong partitioning of clusters.

| **Cluster Stability** | | |
| --- | --- | --- |
| **Unstable Cluster Summary** | **# Patients** | **% Patients** |
| 0 (1,2) | 11 | 73% |
| 1 (1,3) | 2 | 13% |
| 2 (2, 3) | 1 | 7% |
| 3 (1, 2, 3) | 1 | 7% |

5: Hemodynamic parameters on VA ECMO

| **Parameter** |  |
| --- | --- |
| Heart rate (bpm) | 103 ± 16 |
| Systolic blood pressure (mmHg) | 80 ± 8 |
| Diastolic blood pressure (mmHg) | 62 ± 4 |
| Mean arterial blood pressure (mmHg) | 68 ± 3 |
| Arterial pulse pressure (mmHg) | 16 (11-24) |
| Central venous pressure (mmHg) | 9 ± 3 |
| Pulmonary artery systolic pressure (mmHg) | 22 (17-40) |
| Pulmonary artery diastolic pressure (mmHg) | 13 (11-20) |
| Mean pulmonary artery pressure (mmHg) | 15 (12-26) |
| Pulmonary artery pulse pressure (mmHg) | 9 (5-20) |
| End-tidal partial pressure of CO2 (kPa) | 2.9 (2.1-3.6) |
| ECMO flow index (L/min/m^2^) | 2.22 (2.07-2.35) |

6. In-hospital outcomes by aetiology

7. In-hospital outcomes by hemodynamic phenotype

8. Baseline data by sex

| **Parameter** | **Males** | **Females** | **P** |
| --- | --- | --- | --- |
| BMI | 26.2 ± 3.8 | 26.9 ± 4.6 | 0.392 |
| Indication (n, %)  Advanced HF  AMI  Acute myocarditis | 59 (64.8)  22 (24.2)  10 (11) | 23 (62.2)  4 (10.8)  10 (27.0) | 0.036 |
| LVEF | 16 (13-20) | 15 (11-20) | 0.218 |
| LVEDD (cm) | 5.8 (5.1-6.5) | 5.7 (4.5-6.6) | 0.983 |
| TAPSE (mm) | 12 (10-14) | 11 (9-13) | 0.008 |
| RVD (cm) | 3.5 (3.1-3.8) | 3.7 (3.3-4.0) | 0.210 |
| Heart rate (bpm) | 99 (92-108) | 99 (90-110) | 0.380 |
| SBP (mmHg) | 83 (80-88) | 83 (80-86) | 0.994 |
| DBP (mmHg) | 53 (50-56) | 52 (49-55) | 0.234 |
| MAP (mmHg) | 67 ± 4 | 66 ± 3 | 0.347 |
| Lactate (mmol/l) | 6.3 (4.6-9.3) | 5.8 (3.9-9.1) | 0.354 |
| VIS | 20.8 ± 7.0 | 21.4 ± 8.0 | 0.693 |
| Post-ECMO phenotype  LV distension  Low pulsatility  Pulsatile | 25 (27.5)  44 (48.4)  22 (24.2) | 3 (8.1)  22 (59.5)  12 (32.4) | 0.055 |

AMI: acute myocardial infarction; bpm: beats per minute; DBP: diastolic blood pressure; HF: heart failure; LVEDD: left ventricular end-diastolic diameter; LVEF: left ventricular ejection fraction; MAP: mean arterial blood pressure; MR: mitral regurgitation; RVD: right ventricular diameter; SBP: systolic blood pressure; TAPSE: tricuspid annular plane systolic excursion

9. A box plot of ECMO power (the product of VA ECMO flow and MAP) for the three hemodynamic phenotypes. ECMO power was highest in the ‘Pulsatile’ phenotype (p=0.001).


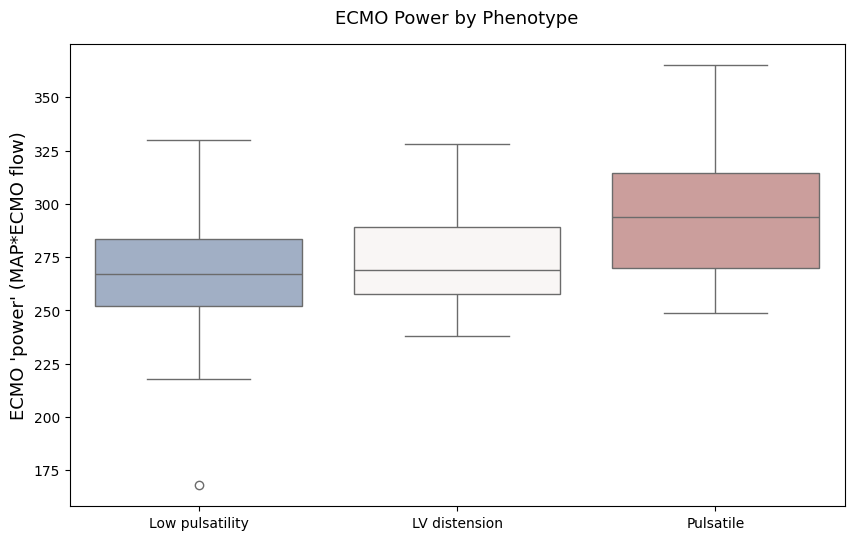

Supplement: Supplementary file 1 — Data S1. [file PHY2-14-e70961-s001.docx]
